# Preventive effects of combinative natural foods produced by elite crop varieties rich in anticancer effects on *N*‐nitrosodiethylamine‐induced hepatocellular carcinoma in rats

**DOI:** 10.1002/fsn3.896

**Published:** 2018-11-29

**Authors:** Jingui Zheng, Jun He, Sufeng Liao, Zuxin Cheng, Jinke Lin, Ke Huang, Xiaocen Li, Kaibin Zheng, Xuanyang Chen, Lihui Lin, Fagang Xia, Jianghong Liu, Ming Xu, Tuansheng Chen, Xinying Huang, Xiaohua Cao, Zhijian Yang

**Affiliations:** ^1^ Agricultural Product Quality Institute Fujian Agriculture and Forestry University Fuzhou China; ^2^ Institute of Laboratory Animal Science Chinese Academy of Medical Sciences Beijing China; ^3^ Anxi College of Tea Science Fujian Agriculture and Forestry University Fuzhou China; ^4^ College of Horticulture and Landscape Hunan Agricultural University Changsha China; ^5^ Institute of Sub‐tropical Agriculture Fujian Academy of Agricultural Sciences Fuzhou China; ^6^ Key Laboratory of Ministry for Education for Genetics, Breeding and Multiple Utilization of Crops Fujian Agriculture and Forestry University Fuzhou China

**Keywords:** anticancer effects, cancer prevention, combinative natural foods, elite crop variety, hepatocellular carcinoma, *N*‐nitrosodiethylamine

## Abstract

The World Cancer Research Fund International has released 32 anticancer effects (ACEs) that targeted every stage of cancer processes. Thus, we designed two formulas of natural food combination Diet I and Diet II, mainly produced by elite crop varieties rich in ACEs with different mixture ratios, and evaluated their cancer preventive effects on *N‐*nitrosodiethylamine (NDEA)‐induced hepatocarcinogenesis. After 20 weeks of dietary intervention, Diet I and Diet II reduced incidence, size, and number of hepatic nodules (*p *<* *0.01) and prevented hepatic tumor formation in NDEA‐induced hepatocarcinogenesis rats. Low‐grade hepatic dysplasia incidence was 20% for Diet II and 40% for Diet I, and apparent hepatocellular carcinomas (HCC) rates were both 0, while 90% HCC in control diet treatment group (*p *<* *0.01). Diet I and Diet II ameliorated abnormal liver function enzymes, reduced serum alpha fetal protein, tumor‐specific growth factor, dickkopf‐related protein 1, tumor necrosis factor‐alpha and interleukin‐6 levels, regulated hepatic phase I and II xenobiotic‐metabolizing enzymes, enhanced antioxidant capacity, suppressed NDEA‐initiated oxidative DNA damage, and induced apoptosis coupled to down‐regulation of proinflammatory, invasion, and angiogenesis markers. Daily intake of combination diet produced from ACEs‐rich elite crop varieties can effectively prevent or delay occurrence and development of NDEA‐induced hepatocarcinogenesis in rats.

## 1 INTRODUCTION

Cancer prevention has been drawing more and more attention in modern society. At the current stage of research, products like synthetic chemical compounds, single natural plant chemicals, and single natural plants are frequently used for cancer prevention. However, most of these chemopreventive agents only target cancer at certain stages of its occurrence and development to suppress tumor development (Kweon, Adhami, Lee, & Mukhtar, 2006; Liu et al., 2017; Stagos et al., 2012; Zhou et al., 2016). This often renders their preventive effects unsatisfactory. For example, for the breast cancer patients who were positive for estrogen receptor, the postoperative recurrence rate decreased by 47% among those who took tamoxifen continuously for 5 years, but the likelihood of those patients developing early endometrial cancer seemed to increase (Buzdar et al., 2006), suggesting that tamoxifen has a serious side effect. According to a report published by Chen, Wallig, and Jeffery (2016), feeding 10% more broccoli (*Brassica oleracea* L. var. *Green Magic*) significantly decreased the levels of hepatic triacylglycerols and tumor necrosis factor, and the rates of nonalcoholic fatty liver disease induced by diethylnitrosamine and diet rich in refined carbohydrate in B6C3F1 mice, but carcinogenesis was not blocked. Yoxall et al. (2005) showed that sulforaphane (SFN) at a typical dietary dose stimulated NAD (P) H quinone dehydrogenase 1 in a dose‐dependent fashion but did not influence glutathione‐S‐transferase (GST), epoxide hydrolase, or uridine diphosphate—glucuronosyl transferase activities in rat livers exposed to SFN in their drinking water for 10 days at equivalent daily doses of 3 and 12 mg/kg.

The 2007 Second Expert Report of the World Cancer Research Fund International (WCRF) has shown that the occurrence and development of cancer are multi‐step processes, and each step can be interrupted by a number of anticancer effects (ACEs) according to Figure 2.5, Chapter 2, such as organic sulfur compounds, epigallocatechin gallate (EGCG), vitamin A, and resveratrol, in total of 32 ACEs (WCRF, 2007) (Supporting Information Table S1).

The concentration of ACEs is crucial to the cancer prevention (Misaka, Miyazaki, Fukushima, Yamada, & Kimura, 2013). In many cases, the effects of chemopreventive agents in cultured cells or tissues are only achievable at supraphysiological concentrations; such concentrations might not be reached when the phytochemicals are administered as part of an organism's diet (Tan, Shi, Tang, Han, & Spivack, 2010). For most people, eating the right foods and drinks is more likely to prevent cancer than dietary supplements, according to the summary of the Third Expert Report of World Cancer Research Fund/American institute for Cancer Research (2018). In our previous work, we have bred and excavated elite crop varieties with high contents of ACEs (Cheng, Xu, Yang, Chen, & Zheng, 2016; Cheng et al., 2014; Zheng, 2004, 2014; Zheng et al., 2012) and the elite crop varieties used in this study as showed in Table 1. For example, broccoli in the market contains a low concentration of SFN, only 0.104 mg/g. We bred a new variety of broccoli, *Brassica oleracea* var. *italica* FU‐1, which contains a high concentration of SFN, 0.984 mg/g (Zheng et al., 2012).

**Table 1:** Content of ACEs in elite crop varieties

Compounds	Crop	Elite crop variety	ACEs contents		References
			Elite crop variety (mg/g)	Common variety (mg/g)	
EGCG	Tea	E‐101	141.3	52.8	Cheng et al. (2016)
Total catechins	Tea	E‐101	224.9	120.1	Cheng et al. (2016)
Sulforaphane	Broccoli	FU‐1	0.984	0.104	Zheng et al. (2012)
Anthocyanins	Black rice	Fuzi No.2	3.096	1.390	Cheng et al. (2016)
	Mulberry	PR‐01	1.93	0.19	Liao et al. (2018)
Procyanidin	Mulberry	PR‐01	155.41	53.53	Cheng et al. (2016)
Selenium	Black rice	Fuzi No.2	7.190 × 10^−5^	1.581 × 10^−5^	Unpublished data
Coixenolide	Job's‐tears	Strain 22	100.8	58.9	Cheng et al. (2016)
Total polyphenol	Job's‐tears	Strain 22	0.762	0.593	Unpublished data
Flavonoid	Job's‐tears	Strain 22	46.85	24.88	Unpublished data
	Mulberry	PR‐01	1.090	0.494	Unpublished data
β‐carotene^a^	Sweet potato	YS‐5	0.090	0.038	Zheng et al. (2012)
	Carrot	Y‐NS	0.021	0.080	Unpublished data
α‐carotene^a^	Carrot	Y‐NS	0.072	0.028	Unpublished data
Lycopene^a^	Carrot	Y‐NS	0.005	0.003	Unpublished data
α‐linolenic acid	Alfalfa	Y‐M551	21.63	6.11	Unpublished data
Resveratrol	Mulberry	PR‐01	0.439	0.017	Cheng et al.(2016)
Polysaccharide	Reishi mushroom	G‐8	72.0	7.40	Cheng et al.(2016)

a The contents of these substances were determined by fresh sample.

In this study, we designed two formulas of natural food combination Diet I and Diet II, which were mainly produced by ACEs‐rich elite crop varieties with different mixture ratios (Supporting Information Table S2) and evaluated the cancer prevention of the two formulas in *N*‐nitrosodiethylamine (NEDA)‐induced hepatocellular carcinoma (HCC) rats. The modulatory effects of Diet I and Diet II on xenobiotic‐metabolizing enzymes, antioxidant activity, and markers of cell proliferation, inflammation, apoptosis, invasion, and angiogenesis during NDEA‐induced rat hepatocarcinogenesis are also explored.

## 2 EXPERIMENTAL SECTION

### 2.1 Chemicals and materials

All chemicals were ordered from Sigma‐Aldrich (St. Louis, MO, USA). TRIzol reagent, HiScript II Q RT SuperMix for qPCR (+cDNA wiper) and AceQTM qPCR SYBRR Green Master Mix were purchased from Takara Co., Ltd. (Dalian, China). Rabbit polyclonal antibodies B‐cell leukemia‐2 (Bcl‐2), Bcl‐2‐associated X protein (Bax), p53, Caspase‐3, and Caspase‐8 were purchased from Santa Cruz Biotechnology; rabbit polyclonal antibodies proliferating cell nuclear antigen (PCNA), nuclear factor‐kappa B (NF‐Κb), vascular endothelial growth factor (VEGF), matrix metalloproteinases 2 (MMP‐2), cyclooxygenase‐2 (COX‐2), tumor necrosis factor‐alpha (TNF‐α), 8‐hydroxydeoxyguanosine (8‐OH‐dG), and matrix metalloproteinases 9 (MMP‐ 9) were purchased from Cell Signaling (Danvers, MA, USA). All other chemicals used were of analytical grade.

### 2.2 Animals, diets, and water

The study was performed in accordance with the Chinese national guidelines for the care of laboratory animals and was approved by the Animal Ethics Committee of the Institute of Laboratory Animal Sciences at the Chinese Academy of Medical Sciences. Five‐week‐old SPF‐grade male Wistar rats were purchased from Beijing Weitong Lihua Experimental Animal Technology Co., Ltd. with animal production license No.: SCXK (Beijing) 2012‐0001, animal certificate number: 11400700135387. All animals were housed in an SPF barrier system of the New Drug Safety Evaluation and Research Center, Institute of Laboratory Animal Sciences, Chinese Academy of Medical Sciences. Breeding conditions were as follows: temperature 22 ± 2°C, relative humidity 50 ± 10%, 12 hr day/night alternation, and access to food and drinking water ad libitum.

The control diet (ConD) was purchased from Beijing Vital River Laboratory Animal Technology Co., Ltd. (Beijing, China). Diet I and Diet II were formulated with energy restriction, and we used natural foods produced by the elite crop varieties (Supporting Information Table S2), which were provided by the Agricultural Product Quality Institute, Fujian Agriculture and Forestry University. The others were purchased from the common market. Diet I is a food combination of nine pure natural foods of plant origin from the elite crop varieties rich in ACEs. 100 g Diet I contains 12 g black rice, 28 g job's‐tears, 10 g sweet potato, 5 g broccoli, 2 g carrot, 1 g alfalfa, 5 g mulberry, and 1.0 g reishi mushroom and is supplemented with 7 g corn, 24 g bean cake, 1 g dried yeast, 2 g bran, 1.0 g oleum morrhuae and then blended to mixture. The mixture was then grain shaped, dried at 60°C and sterilized by Co^60^. Diet II is composed of primarily by foods of nine pure natural foods of plant origin from the elite crop varieties rich in ACEs and subsidiary by three light meat. 100 g Diet II contains 11.5 g black rice, 26.9 g job's‐tears, 7.7 g sweet potato, 4.8 g broccoli, 1.9 g carrot, 0.5 g alfalfa, 4.8 g mulberry, and 1.4 g reishi mushroom, 3.8 g trepang, 1.0 g abalone, 1.4 g clam and is supplemented with 5.3 g corn, 16.3 g bean cake, 1.4 g dried yeast, 1.9 g bran, 1.0 oleum morrhuae, 1.9 g grifola frondosa, 1.4 g kiwi fruit, 1.4 g pollen, and 1.4 g fig and then blended to mixture. The mixture was then grain shaped, dried at 60°C and sterilized by Co^60^.

The EGCG‐rich tea [*Camellia sinensis* (L.) O. Kuntze] variety E‐101 was processed as following: tea powder (30 g) was extracted with 1 L distilled water at 80°C, steeped for 5 min, ultrasonic extraction for 10 min with Power of 200 W, two rounds of extraction, combined filtration, then collection of the E‐101 tea water.

### 2.3 Experimental design

After 1‐week adaptive feeding on control diet, forty rats were randomly divided into four groups (*n* = 10/group): normal control group (ConD group), model group (ConD+NDEA group), Diet I+NDEA group, and Diet II+NDEA group. From the first week of experiment, rats in ConD and ConD+NDEA groups were fed with the control diet and offered water ad libitum. The rats in Diet I+NDEA and Diet II+NDEA group were fed with Diet I or Diet II, and both drank E‐101 tea water instead of drinking water for 20 weeks. In the 2–14th week of the experiment, rats in the model group, Diet I+NDEA group, and Diet II+NDEA group were with 25 mg/kg NDEA (twice a week) as described earlier (Liao, Liu, Xu, & Zheng, 2018). Rats in the ConD group received intraperitoneal injection of the same volume of saline. Body weights, feed and tea, or water intake were measured weekly. After 20 weeks, the rats were anesthetized by intraperitoneal injection of pentobarbital sodium (40 mg/kg) for collection of blood samples. Immediately after that, animals were sacrificed by bleeding from the abdominal aorta, and their organs were collected. The size and number of liver nodules were measured as described earlier (Chen et al., 2016). Liver tissue was collected for biochemical, histopathological, and ultrastructural analyses.

### 2.4 Histological analysis of the liver tissue

Small blocks of liver from median lobe were fixed in 10% neutral buffered formalin, embedded in paraffin, sectioned (4 μm), and stained with hematoxylin and eosin (H&E) for histopathological examination. The stained slides were analyzed by assessing the morphological changes under the OPTIPHOT‐2 light microscope by an experienced investigator that was unaware of the experimental conditions. Five microscopy‐stained slides per animal were examined. The extent of cancerization was assessed according to Edmondson classification method (Edmondson & Steiner, 1954).

For liver electron microscopy, pinhead‐sized blocks of liver (1 mm × 1 mm × 1 mm) was fixed in 2.5% glutaraldehyde in 0.1 M Sodium cacodylate buffer (pH 7.4) for 1 hr at 4°C and postfixed in 1% osmium tetroxide. Ultrathin sections (90 nm) were cut on MT‐500 ultramicrotome (DuPont, USA), mounted on 300 mesh copper grids, stained with uranyl acetate and lead citrate, and examined under JEM‐1400 transmission electron microscope (JEOL, Japan).

### 2.5 Biochemical assays

The levels of serum aspartate transaminase (AST), Alanine aminotransferase (ALT), GST, γ‐glutamyl transpeptidase (GGT), total bilirubin (TBIL), alkaline phosphatase (ALP), total protein (TP), and albumin (ALB) were detected according to the instructions of commercial kits (Leadman Biochemistry Co., Ltd., Beijing, China) on Hitachi 7100 automatic biochemical analyzer (Hitachi, Ltd., Tokyo, Japan). The levels of alpha fetal protein (AFP), tumor‐specific growth factor (TSGF), dickkopf‐related protein 1 (DKK1), tumor necrosis factor‐alpha (TNF‐α), carcinoembryonic antigen (CEA), and interleukin‐6 (IL‐6) were detected using ELISA kit (CUSABIO Biotech Co., Ltd., Wuhan, China) according to manufacturer's instructions. The inflammatory parameters of white blood cell, neutrophil, and lymphocyte counts were measured on the Pentra DX 120 automatic blood analyzer (ABX, France).

Microsomal fractions were prepared from liver tissues as described previously (Velayutham et al., 2007). The concentration of microsomal protein was determined using the BCA kit. The contents of cytochrome b5 (Cyt b5) and cytochrome P450 (Cyt P450) were assayed using the method published by Omura and Sato (1964). The activity of NADPH‐cytochrome b5 reductase (Cyt b5R) was assayed using the methods published by Philips and Langdon (1962). Cytochrome C (Cyt c) was used as the substrate to determine NADH‐cytochrome P450 reductase (Cyt P450R) and DT‐diaphorase (DTD) activity using the methods reported by Cummings, Parker, and Lash (2000) and Smitskamp‐Wilms, Giaccone, Pinedo, vander, and Peters (1995), respectively. Aryl hydrocarbon hydroxylase (AHH) and aniline hydroxylase CYP2E1 (ANH) activity were determined using double‐beam ultraviolet spectrophotometry (Nebert & Gelboin, 1968). The activities of GST and UDP‐glucuronosyltransferase (UGT) in the liver homogenates were detected by specific ELISA kits (Shanghai Jiang Lai Biotechnology Co., Shanghai, China). The epoxide hydrolyzyme (EPT) activity in liver microsome was determined using the Fabian method (Fabian et al., 2016).

The malondialdehyde (MDA) content and the activities of superoxide dismutase (SOD), catalase (CAT), and glutathione peroxidase (GSH‐Px) were assayed within 12 hr using standard commercially available kits according to the manufacturer's instructions (Nanjing Jiancheng Bioengineering Engineering Institute, Nanjing, China).

### 2.6 Immunohistochemical analysis

Paraffin section (4 μm) was dewaxed and rehydrated through a gradual decrease in ethanol concentration. The slides were incubated in sodium citrate buffer for two cycles of 5 min at 37°C for antigen retrieval. After washing with phosphate buffer solution (PBS), the slides were then treated with 3% hydrogen peroxide to remove any endogenous peroxidases. Then the sample was washed with PBS for three cycles of 5 min and blocked with normal goat serum in a humidified chamber for several hours at 4°C. The sections were then incubated overnight at 4°C in a humidified chamber using the appropriate primary antibodies: 8‐OH‐dG, NF‐κB, COX‐2, TNF‐α, p53, Bcl‐2, PCNA, and VEGF with recommended dilution. The slides were washed with TBS and then incubated with HRP‐labeled sheep antirabbit secondary antibody at room temperature for 1 hr followed by streptavidin‐biotin‐peroxidase at room temperature for 30 min. The slides were washed with PBS, and the immunoprecipitation was visualized by treating with 3, 3′‐diaminobenzidine for color development for 25 min. Then slides were counterstained with hematoxylin, and the brown color signifying the presence of antigen bound to antibody was detected by light microscopy. For the negative control, tris‐buffered saline (TBS) was used instead of a primary antibody. From ten randomly selected sections of each slide, 500 cells were counted. The percentage of positive cells for each group was calculated using the Image‐Pro Plus 6.0 image analysis system.

### 2.7 Quantitative real‐time PCR assay

Total RNA was isolated from liver tissue using TRIzol (Invitrogen). The cDNA was synthesized from the RNA samples. The real‐time quantitative polymerase chain reaction (qRT‐PCR) reaction was carried out using the SYBR^®^ Fast qPCR Mix (Code no. RR430A, TaKaRa, Japan) via the Real‐time PCR System (ABI Prism 7500, Life Technology, MA, USA). The qPCR reaction conditions were as follows: 95°C for 15 s, annealing at 60°C for 10 s, and extension at 72°C for 34 s for a total of 40 cycles. The primer sequences were shown in Supporting Information Table S3. GAPDH served as an internal control for all measurements. Each sample was amplified in triplicate. Relative gene expression data were analyzed using the 2^−ΔΔCT^ method (Sur et al., 2016).

### 2.8 Western blot analysis

The protein was extracted from liver tissue with RIPA buffer containing protease inhibitor cocktail. The protein extraction was separated using centrifugation (15,000 × *g*; 15 min; 4°C), and the supernatant was collected and then quantified using the BCA kit. Equal amounts of proteins were separated through 10%–12% SDS‐polyacrylamide electrophoresis gels and transferred to polyvinylidene fluoride membranes. After blocked with 5% skim milk, the membranes were incubated with the appropriate primary antibodies respectively, and β‐actin (CUSABIO Biotech Co., Ltd., Wuhan, China) with recommended dilution at 4°C overnight, and then incubated with goat antirabbit IgG horse radish peroxidase (HRP) secondary antibodies at room temperature for 1 hr. The films were developed using an ECL Plus chemiluminescence reagent kit (Millipore, MA, USA) and visualized using ChemiDocXRS (Bio‐Rad Laboratory, CA, USA). Densitometries were analyzed using Quantity One software and normalized to β‐actin.

### 2.9 Statistical analysis

Statistical analysis was performed using SPSS19.0 software. All results are expressed as mean ± standard deviation (SD) and were analyzed using a one‐way, 2‐ or 3‐factor ANOVA, followed by LSD test or Tukey's test when the differences were indicated. A *p* value of <0.05 was here considered significant in this study.

## 3 RESULTS

### 3.1 Body weight, relative liver weight, feed and tea/water intake were not changed by long‐term Diet I and Diet II consumption

In NDEA administered rats, the food intake, water consumption, and body weight gain were significantly (*p *<* *0.05) decreased (Supporting Information Table S4) and the relative liver weight was significantly (*p *<* *0.01) increased in NDEA+ConD group rats relative to ConD group rats (Supporting Information Table S4). All the changes induced by NDEA intoxication were significantly (*p *<* *0.01 or *p *<* *0.05) reduced except body weight when rats treated with Diet I and Diet II (Supporting Information Table S4), and there were no significant changes in hematological indicators (Supporting Information Table S5).

### 3.2 Diet I and Diet II exerted a powerful protective or delaying effect on hepatic tumorigenesis

When treated with NDEA (ConD+NDEA group), 100% rats developed nodules in the liver (for the two rats died of severe hepatic tumor pathogenesis at week 16 and 19 respectively, and the remaining eight rats, the nodule formation was observed. While administration of Diet I (Diet I+NDEA group) and Diet II (Diet II+NDEA group) was found to be associated with marked decreases in the number and multiplicity of the nodules relative to ConD +NDEA group rats (*p *<* *0.01, Table 2). The incidence of nodule growth was reduced to 30% in the Diet I+NDEA group and 50% in the Diet II+NDEA group, respectively (Table 2). No hepatic nodules were observed in the ConD group animals.

**Table 2:** Effects of Diet I and Diet II on hepatic neoplasm‐related lesions in NDEA‐induced hepatocarcinogenesis rats at 20th week

Parameters	ConD	ConD+NDEA	Diet I+NDEA	Diet II+NDEA
Mortality (%)	0/10 (0.0)	2/10 (20.0)*	0/10 (0.0)#	0/10 (0.0)#
Relative liver weight^a^	2.49 ± 0.20	3.59 ± 0.34**	3.23 ± 0.31*	2.86 ± 0.27##
Macroscopic lesions				
Nodule incidence (%)	0/10 (0.0)	8/8 (100.0)**	3/10 (30.0)##	5/10 (50.0)#
Total number of nodules (n)	0	834**	3##	6##
Nodulemultiplicity (n)^b^	0	83.4 ± 61.9**	0.3 ± 0.5##	0.6 ± 0.7##
<1 mm	—	281 (42.1)	2 (66.7)	3 (60.0)
>1 mm <3 mm	—	238 (35.7)	1 (33.3)	2 (40.0)
>3 mm	—	148 (22.2)	0 (0.0)	0 (0.0)
Max nodule diameter(mm)	—	23.38 ± 14.52	1.00 ± 0.50##	1.40 ± 0.89##
Tumor volume (mm^3^)/rat	—	99.70 ± 90.51	0.10 ± 0.30##	6.80 ± 1.60##
Microscopic lesions				
Low‐grade dysplasia incidence (%)	0/10 (0.0)	0/10 (0.0)	4/10 (40.0)#	2/10 (20.0)
Hepatic adenoma incidence (%)	0/10 (0.0)	1/10 (10.0)	0/10 (0.0)	0/10 (0.0)
HCC incidence (%)	0/10 (0.0)	9/10 (90.0)**	0/10 (0.0)##	0/10 (0.0)##
Liver metastases (%)	0/10 (0.0)	2/10 (20.0)	0/10 (0.0)	0/10 (0.0)

^a^Relative liver weight = liver weight/body weight. ^b^Average number of nodules/nodules bearing liver. Data are means ± *SD* or *n* (%), *n* = 8–10. Comparisons: compared with ConD group, *p <* *0.05; **p <* *0.01; compared with ConD+NDEA group, ^#^ *p *<* *0.05; ^##^ *p *<* *0.01 (Fisher's exact test). ConD: normal control group; ConD+NDEA: model group, NDEA plus control diet‐treated group; Diet I+NDEA: NDEA plus Diet I‐treated group; Diet II+NDEA: NDEA plus Diet II‐treated group.

**Figure 1:**
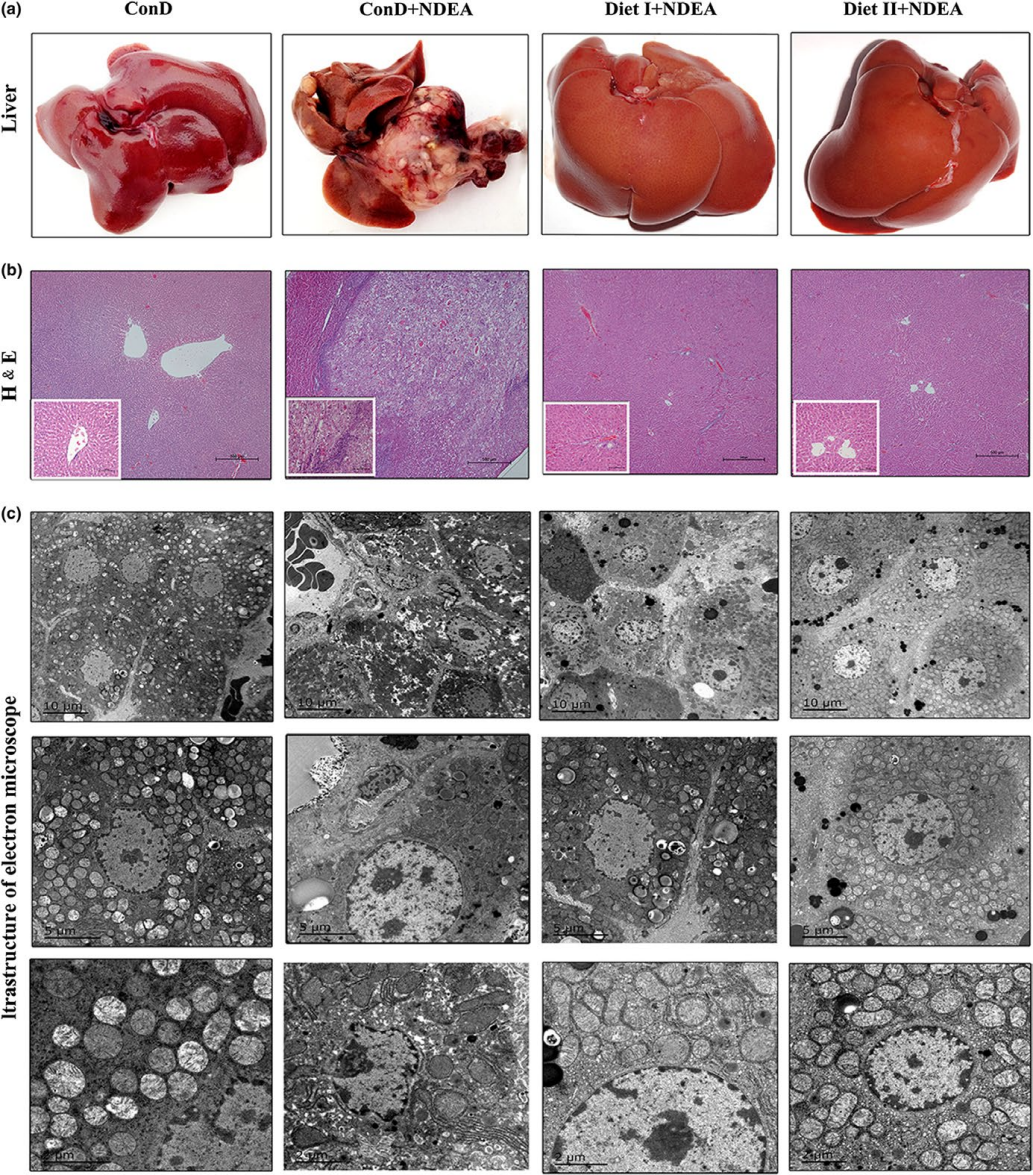
Effects of Diet I and Diet II on liver tissue pathological changes in NDEA‐induced hepatocarcinogenesis rats. (a) Representative images of the livers from each group rats. (b) Representative liver histopathological changes using hematoxylin and eosin (H&E) staining of rats are shown as ×40 magnifications (big figure) and ×200 magnifications (small figure). (c) Representative transmission electron microscope (EM 2000) images of changes in the ultrastructure of hepatic cells in rats. ConD: normal control group; ConD+NDEA: model group, NDEA plus control diet‐treated group; Diet I+NDEA: NDEA plus Diet I‐treated group; Diet II+NDEA: NDEA plus Diet II‐treated group. ConD: control diet; NDEA:* N*‐nitrosodiethylamine

The results of H&E staining (Figure 1B and Table 2) showed normal hepatocyte structure in the ConD group rats and clustered distribution of tumor cells in the liver tissues of ConD+NDEA group rats. The oval cells and bile duct hyperplasia were readily visible with 90% incidence of apparent HCC in the model group. Administration of Diet I and Diet II associated with a marked decrease in the incidence of hepatic adenoma or HCC formation (with 0 incidence of apparent HCC in the Diet I+NDEA and Diet II+NDEA groups compared to the 90% incidence of apparent HCC in the model group*, p *<* *0.01), and only a few inflammatory cells were observed during histopathological analysis. The morphological structure of the rat hepatocytes in the Diet II+NDEA group was similar to that in normal liver tissues (Figure 1b), and the low‐grade hepatic dysplasia incidence was 20% and 40% in the Diet I+NDEA group (Table 2). Further electron microscopy (Figure 1c) showed that the hepatocytes in the model group were not of uniform size, had increased nuclear volume with mega or double nuclei, swollen mitochondria with large vacuoles and significant reductions in the number of mitochondria relative to the normal control group. Compared with the ConD+NDEA group, the number and size of hepatocyte lesions were significantly lower in the Diet I+NDEA group, and the hepatocyte structure of the Diet II+NDEA group was similar to that of normal cells. These results indicated that Diet I and Diet II could prevent or delay the progress of NDEA‐induced hepatocarcinogenesis and significantly alleviated the hepatocellular precancerous lesions in rats.

### 3.3 Diet I and Diet II ameliorated hepatocyte damage and decreased serum tumor markers

Detection of blood biochemical indicators showed that the levels of liver function markers, such as AST, ALT, GGT, TBIL, ALP, and albumin in serum of ConD+NDEA group rats were significantly higher than in the ConD group (Table 3), indicating that the stimulation of NDEA and its metabolites in vivo on hepatocytes caused severe injury and liver dysfunction in the ConD+NDEA group. In the presence of Diet I and Diet II administration, the activities of the above serum marker enzymes in the serum of Diet I+NDEA and Diet II+NDEA group rats were significantly lower than in the ConD+NDEA group (Table 3), which was similar to the normal control group. The levels of AFP, TSGF, CEA, and DKK1 were much higher in the ConD+NDEA group than in the normal control group (*p *<* *0.01); and Diet I and Diet II significantly inhibited the elevation of the serum tumor markers, AFP, TSGF, and DKK1 (*p *<* *0.01, Table 3).

**Table 3:** Effects of Diet I and Diet II on blood plasma biochemistry in NDEA‐induced hepatocarcinogenesis rats at 20th week

Parameter/Groups	ConD	ConD+NDEA	Diet I+NDEA	Diet II+NDEA
AST(U/L)	65.00 ± 36.33	185.63 ± 61.70**	137.10 ± 4.52**##	99.20 ± 13.50*##
ALT(U/L)	40.70 ± 8.84	107.25 ± 42.48**	40.30 ± 11.76##	35.10 ± 9.48##
GGT(U/L)	1.61 ± 0.24	2.57 ± 0.21**	1.57 ± 0.62##	1.52 ± 0.12##
TBIL(μmol/L)	2.32 ± 0.26	4.20 ± 0.94**	2.24 ± 0.40##	2.30 ± 0.17##
ALP(U/L)	56.60 ± 10.43	85.63 ± 17.11**	62.20 ± 20.82##	53.80 ± 9.37##
Total protein (g/L)	65.75 ± 9.78	65.60 ± 12.25	59.37 ± 9.46	60.43 ± 10.83
Albumin (g/L)	27.04 ± 0.83	29.26 ± 2.11*	25.38 ± 2.50##	26.76 ± 2.12##
Globulin (g/L)	38.71 ± 10.12	36.34 ± 11.43	29.99 ± 9.94	33.67 ± 10.83
AFP(ng/ml)	3.73 ± 0.50	5.84 ± 1.26**	3.25 ± 0.54##	3.53 ± 1.67##
CEA(ng/ml)	1.83 ± 0.34	2.25 ± 0.34**	2.07 ± 0.30	2.08 ± 0.38
TSGF(μg/ml)	23.70 ± 1.34	36.31 ± 7.99**	28.39 ± 5.14##	26.09 ± 4.79##
DKK1(ng/ml)	1.47 ± 0.23	7.83 ± 1.85**	3.61 ± 0.90##	3.20 ± 1.20##

Values are given as mean ± *SD*,* n* = 8. Comparisons: compared with ConD group, *p <* *0.05; **p <* *0.01; compared with ConD+NDEA group,^##^ *p *<* *0.01 (Student's *t* test). AFP: alpha fetal protein; ALB: albumin; ALP: alkaline phosphatase; ALT: alanine aminotransferase; AST: aspartate transaminase; CEA: carcinoembryonic antigen; DKK1: dickkopf‐related protein 1; GGT: γ‐glutamyl transpeptidase; GST: glutathione‐S‐transferase; TBIL: total bilirubin; TP: total protein; TSGF: tumor‐specific growth factor.

### 3.4 Diet I and Diet II suppressed the activity of phase I enzymes and enhanced that of phase II enzymes

Carcinogens are primarily activated by phase I enzymes. The body can be protected from carcinogens by induction of phase II enzymes that lead to detoxification and accelerated excretion of carcinogens (Chakraborty et al., 2007; Na & Surh, 2008). As shown in Table 4, NDEA treatment significantly increased the concentrations of Cyt b5, Cyt P450 and the activities of Cyt b5R, Cyt P450R, DTD, and AHH in livers of ConD+NDEA group rats compared to the normal control group rats (all *p *<* *0.01) and significantly reduced the activities of liver phase II enzymes such as GST, UGT, and EPT (all *p *<* *0.01). Administration of Diet I or Diet II to NDEA‐treated animals significantly reduced the concentrations of Cyt b5 and Cyt P450 and suppressed the activities of Cyt b5R, Cyt P450R, DTD, and AHH in the livers (all *p *<* *0.01), significantly increased the activities of GST, UGT, and EPT in livers over rats treated with NDEA alone (all *p *<* *0.01) (Table 4). These results demonstrated that Diet I and Diet II act as dual‐acting agent by suppressing phase I enzymes and enhancing phase II enzyme activity, thereby promoting detoxification and excretion.

**Table 4:** Effect of Diet I and Diet II on Phase I and II enzymes in the livers of NDEA‐induced hepatocarcinogenesis rats

Parameters	ConD	ConD+NDEA	Diet I+NDEA	Diet II+NDEA
Phase I enzymes				
Cyt b5(nmoles/mg of protein)	0.26 ± 0.03	0.50 ± 0.06**	0.32 ± 0.05##	0.28 ± 0.04##
Cyt P450(nmoles/mg of protein)	0.34 ± 0.03	0.99 ± 0.11**	0.52 ± 0.07**##	0.42 ± 0.06##
Cyt b5R (nmoles/min/mg of protein)	1.12 ± 0.07	1.91 ± 0.16**	1.45 ± 0.19*##	1.36 ± 0.20##
Cyt P450R(cyt c reduced/min/mg of protein)	1.13 ± 0.10	2.04 ± 0.37**	1.49 ± 0.19##	1.40 ± 0.17##
AHH(nmoles of 3‐OHBaP formed/min/mg of protein)	4.84 ± 0.16	8.79 ± 1.44**	5.23 ± 1.39##	5.40 ± 1.23##
DTD(cyt c reduced/min/mg of protein)	1.57 ± 0.11	2.36 ± 0.42**	1.72 ± 0.35##	1.63 ± 0.27##
ANH(nMp‐aminophenol/min/mg protein)	1.39 ± 0.12	1.28 ± 0.22	1.55 ± 0.19	1.53 ± 0.20
Phase II enzymes				
GST(μmol/min/mg protein)	7.14 ± 1.16	4.14 ± 0.57**	6.24 ± 1.25##	6.30 ± 1.21##
UGT(μmol/min/mg protein)	15.38 ± 0.79	6.48 ± 2.14**	12.75 ± 1.95##	11.13 ± 3.26*##
EPT(μmol/min/mg protein)	4.34 ± 0.23	2.61 ± 0.33**	4.80 ± 0.47*##	4.49 ± 0.45##

Values are expressed as mean ± *SD* (*n* = 8). Comparisons: compared with ConD group, *p <* *0.05; **p <* *0.01; compared with ConD+NDEA group, ^##^ *p *<* *0.01. 3‐OHBaP: 3‐ hydroxybenzo (a) pyrene; AHH: Aryl hydrocarbon hydroxylase; ANH: aniline hydroxylase CYP2E1; Cyt b5: cytochrome b5; Cyt b5R: NADPH‐cytochrome b5 reductase; Cyt c: Cytochrome C; Cyt P450: cytochrome P450; Cyt P450R: NADH‐cytochrome P450 reductase; DTD: DT‐diaphorase; EPT: epoxide hydrolyzyme; GST: glutathione‐S‐transferase; UGT: UDP‐glucuronosyltransferase.

### 3.5 Diet I and Diet II reduced NDEA‐induced oxidative stress

Oxidative stress is one of the major instigators of the pathogenesis of environmental cancer (Grace et al., 2016). NDEA is converted to reactive oxygen species in liver cells through phase I enzyme metabolism, causing oxidation and inducing oxidative damage to DNA, ultimately leading to pathological changes and initiation of hepatogenesis (Marra et al., 2011). Many chemical carcinogens are associated with free radicals or reactive oxygen species, such as O^2−^, H_2_O_2_, and ROOH, and scavenging this free radical or reactive oxygen is mainly dependent on SOD, CAT, GSH‐Px, and GSH (Bishayee et al., 2011). As shown in Table 5, single intraperitoneal administration of NDEA significantly elevated serum and liver MDA content (*p *<* *0.01) and inhibited the activities of SOD, GSH‐Px, and CAT in serum and livers of ConD+NDEA group as compared to the normal controls (all *p *<* *0.01). However, when fed with Diet I or Diet II for 20 weeks, the activities of SOD, GSH‐Px, and CAT in serum and livers of the Diet I+NDEA or Diet II+NDEA group rats were significantly higher (*p *<* *0.01 or *p *<* *0.05) than those of ConD+NDEA group. MDA content was also significantly lower as compared to ConD+NDEA group (*p *<* *0.01).

**Table 5:** Effects of Diet I and Diet II on antioxidant activity in the plasma and livers of rats with NDEA‐induced hepatocarcinogenesis

Parameters	ConD	ConD+NDEA	Diet I+NDEA	Diet II+NDEA
Serum				
SOD (U/ml)	40.92 ± 3.64	26.36 ± 5.19**	46.41 ± 5.05##	49.99 ± 5.83##
GSH‐Px (U/ml)	465.53 ± 24.22	363.99 ± 43.51**	437.94 ± 40.80##	458.47 ± 22.20##
CAT (U/ml)	21.42 ± 1.70	12.37 ± 1.89**	18.95 ± 2.85##	19.80 ± 2.32##
MDA (nmol/ml)	4.81 ± 1.77	9.59 ± 2.16**	4.98 ± 1.62##	5.80 ± 1.32##
Liver				
SOD(U/mg)	10.64 ± 1.56	7.43 ± 0.52**	8.89 ± 2.19#	9.62 ± 0.99##
GSH‐Px (U/mg)	116.03 ± 7.24	89.26 ± 10.94**	112.39 ± 8.11#	123.93 ± 7.58##
CAT (U/mg)	2.77 ± 0.77	1.46 ± 0.65**	2.07 ± 1.03#	2.87 ± 0.46##
MDA (nmol/mg)	1.38 ± 0.36	2.68 ± 0.84**	1.54 ± 0.36##	1.60 ± 0.31##

Values are expressed as mean ± SD (*n* = 8). Comparisons: compared with ConD group, **p <* *0.01; compared with ConD+NDEA group, ^#^ *p *<* *0.05; ^##^ *p *<* *0.01. CAT: catalase; GSH‐Px: glutathione peroxidase; MDA: malondialdehyde; SOD: superoxide dismutase.

**Figure 2:**
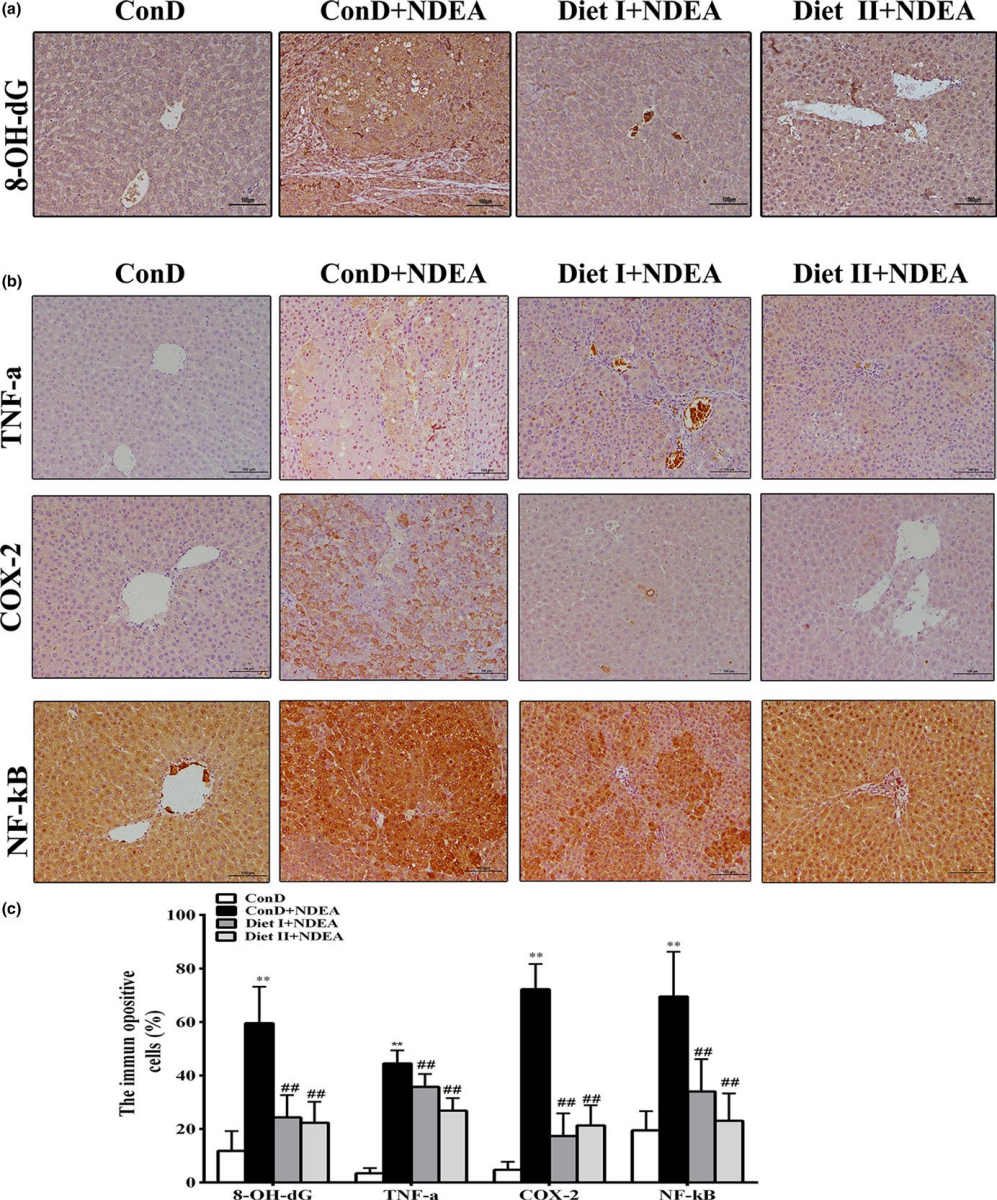
Effects of Diet I and Diet II on biomarker of oxidative DNA damage and inflammation in liver of NDEA‐induced hepatocarcinogenesis rats by immunohistochemical staining. (a) Representative immunohistochemical staining and average percentage of 8‐OH‐dG positive stained cells in the liver of control and experimental animals at ×200 original magnification. (b) Representative immunohistochemical staining of TNF‐α, COX‐2, and NF‐κB positive stained cells in the liver of control and experimental animals at ×200 original magnification. (c) The average percentage of TNF‐α, COX‐2, and NF‐κB positive stained cells in the liver of control and experimental animals. Values are expressed as mean ± *SD* (*n* = 6). Comparisons: compared with the ConD group, **p <* *0.01, *p <* *0.05; compared with the ConD+NDEA group, ^#^ *p *<* *0.05; ^##^ *p *<* *0.01. 8‐OH‐dG: 8‐hydroxydeoxyguanosine; NF‐κB: nuclear factor‐kappa B; COX‐2: cyclooxygenase‐2; TNF‐α: tumor necrosis factor‐alpha

The 8‐OH‐dG, as a biomarker of oxidative DNA damage, was detected using immunohistochemistry to evaluate oxidative DNA damage in the liver. As shown in Figure 2a, the production of 8‐OH‐dG increased significantly in the liver tissue of ConD+NDEA rats (*p *<* *0.01), indicating severe DNA damage in hepatocytes. However, the production of 8‐OH‐dG in liver tissue of rats fed with Diet I or Diet II was significantly lower than the level of production in the ConD+NDEA group (*p *<* *0.01). These results showed that Diet I and Diet II can reduce NDEA‐induced oxidative stress and protect the body from oxidative DNA damage.

### 3.6 Diet I and Diet II can reduce NDEA‐induced inflammatory response

**Figure 3:**
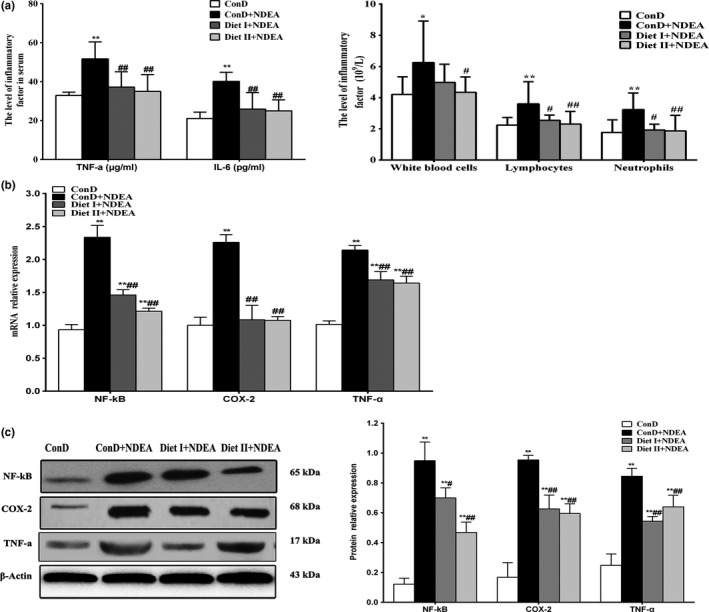
Effects of Diet I and Diet II on markers of inflammation in liver of NDEA‐induced hepatocarcinogenesis rats. (a) The levels of TNF‐α, IL‐6, and leukocyte count, neutrophil and lymphocyte count in blood. (b) The mRNA relative expression levels of NF‐κB, COX‐2, and TNF‐α in control and experimental groups. GAPDH was used as an internal control. (c) Representative immunoblots of NF‐κB, COX‐2, and TNF‐α. β‐actin was used as an internal control. Values are expressed as mean ± *SD* (*n* = 3‐6). Comparisons: compared with the ConD group, **p <* *0.01, *p <* *0.05; compared with the ConD+NDEA group, ^#^ *p *<* *0.05; ^##^ *p *<* *0.01. TNF‐α: tumor necrosis factor‐alpha; IL‐6: interleukin‐6; NF‐κB: nuclear factor‐kappa B; COX‐2: cyclooxygenase‐2

NDEA may cause severe chronic inflammatory damage to the liver, thus triggering the development and progression of HCC (Raghunandhakumar et al., 2013). Inflammatory factors (TNF‐α, IL‐6, etc.) play important roles in the development of inflammation and tumorigenesis (Sivaramakrishnan & Niranjali, 2009; Sui et al., 2014; Wang et al., 2017; Wojcik et al., 2012). The interaction between TNF‐α, IL‐6, and NF‐κB initiates a vicious cycle in the cytokine network, and TNF‐α and IL‐6 may activate NF‐κB, further amplifying the inflammatory response. Activated NF‐κB may further induce or activate COX‐2 (Wang et al., 2017). The mRNA and protein expression of NF‐κB, COX‐2, and TNF‐α are presented in Figures 2, 3. As shown in Figure 3a, the levels of TNF‐α and IL‐6 in the serum of the model group were significantly higher than in the normal control group (*p *<* *0.01), as well as the number of white blood cells, neutrophils, and lymphocytes (*p *<* *0.01 or *p *<* *0.05). The mRNA and protein expression of NF‐κB, COX‐2, and TNF‐α in the liver of ConD+NDEA group were also significantly increased (*p *<* *0.01 or *p *<* *0.05). These results suggest that NDEA‐induced hepatocarcinogenesis may be associated with the inflammatory response. The Diet I+NDEA and Diet II+NDEA groups showed significantly lower serum TNF‐α and IL‐6 levels than in the ConD+NDEA group (*p *<* *0.01 or *p *<* *0.05), significantly lower number of white blood cells, neutrophils and lymphocytes (*p *<* *0.01 or *p *<* *0.05, except for white blood cell count in the Diet I +NDEA group), and significantly lower mRNA and protein expression of NF‐κB, COX‐2, and TNF‐α in liver (*p *<* *0.01). These results suggest that Diet I and Diet II can inhibit NDEA‐induced systemic inflammatory response, reduce the accumulation of inflammatory factors, thus having an optimal regulatory function against pathological changes in the liver and preventing the occurrence or delaying development of cancer.

### 3.7 Diet I and Diet II induced cell apoptosis and inhibited tumor cell proliferation, angiogenesis, and invasion

NDEA causes genomic damage in exposed cells. This can trigger the damaged cells to proliferate, leading to the formation of cancerous cells, which showed increased cell proliferation, angiogenesis, and invasion potential (Hanahan & Weinberg, 2011; Yu et al., 2012).

**Figure 4:**
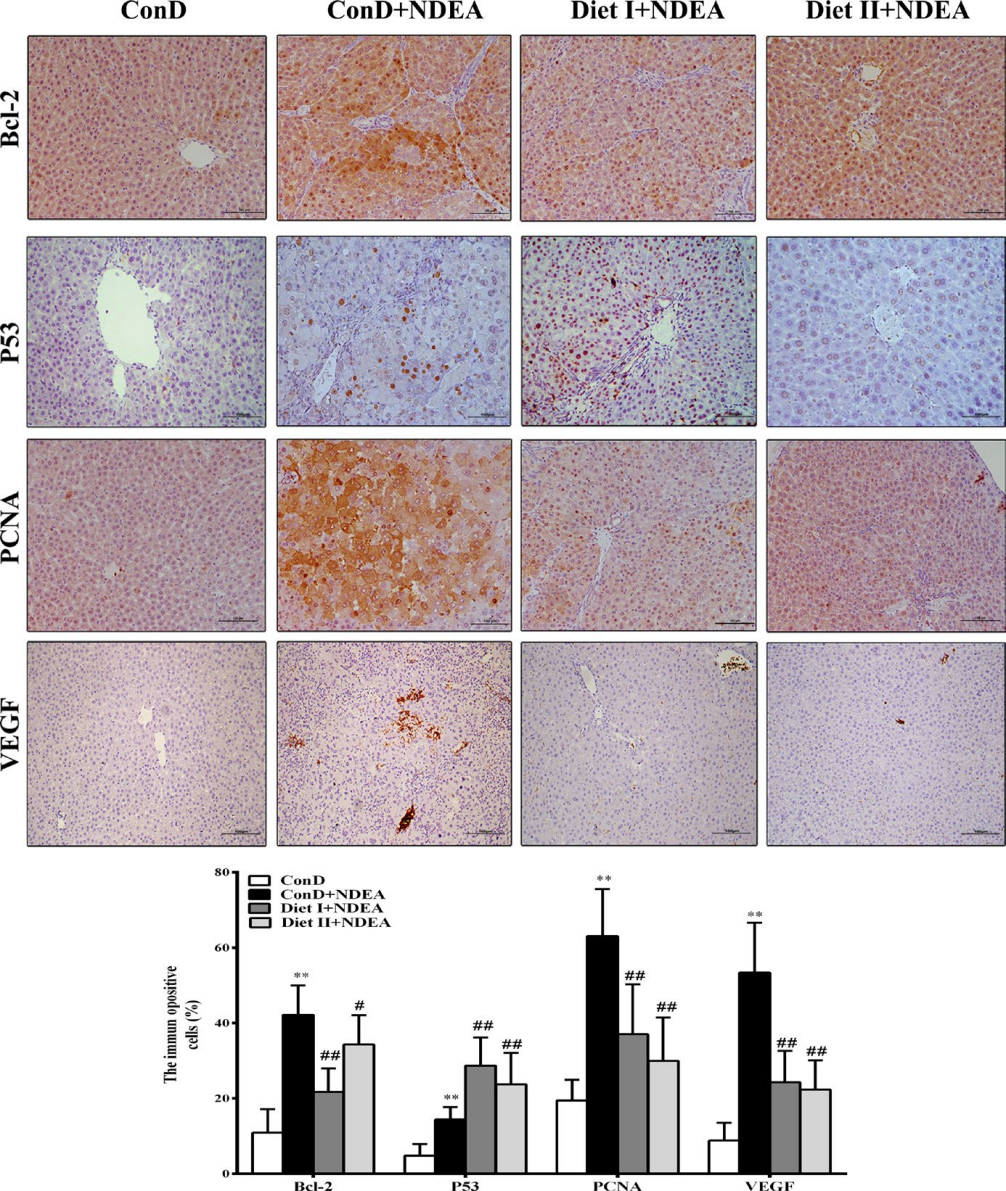
Effect of Diet I and Diet II on the expression of Bcl‐2, P53, PCNA, and VEGF in livers of NDEA‐induced hepatocarcinogenesis rats by immunohistochemical staining. Bar graph represents average percentage of positive stained cells of Bcl‐2, P53, PCNA, and VEGF in the livers of control and experimental groups. Values are expressed as mean ± *SD* (*n* = 6). Comparisons: compared with the ConD group, **p <* *0.01, *p <* *0.05; compared with the ConD+NDEA group, ^#^ *p *<* *0.05; ^##^ *p *<* *0.01. Bcl‐2: B‐cell leukemia‐2; Bax: Bcl‐2‐associated X protein; PCNA: proliferating cell nuclear antigen; VEGF: vascular endothelial growth factor; MMP‐2: matrix metalloproteinases 2; MMP‐9: matrix metalloproteinases 9

**Figure 5:**
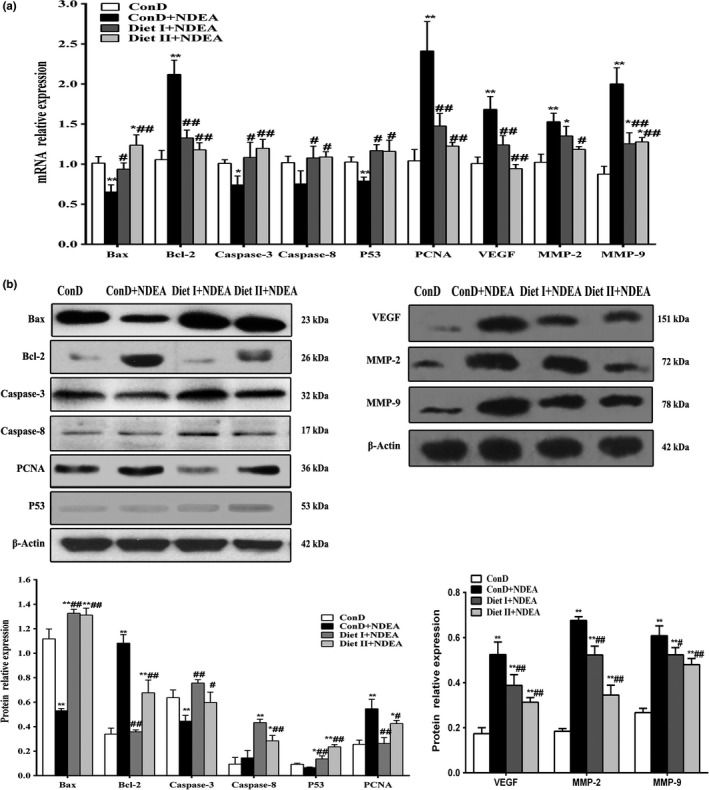
Effect of Diet I and Diet II on the markers of apoptosis, proliferation, angiogenesis, and invasion in livers of NDEA‐induced hepatocarcinogenesis rats by western blot and qRT‐PCR analyses. (a) mRNA relative expression levels of Bax, Bcl‐2, Caspase‐3, Caspase‐8, p53, PCNA, VEGF, MMP‐2, and MMP‐9 in control and treatment groups; GAPDH was used as an internal control. (b) Representative immunoblots of Bax, Bcl‐2, Caspase‐3, Caspase‐8, PCNA, and p53; β‐actin was used as an internal control. Values are expressed as mean ± *SD* (*n* = 3–6). Comparisons: compared with the ConD group, **p <* *0.01, *p <* *0.05; compared with the ConD+NDEA group, ^#^ *p *<* *0.05; ^##^ *p *<* *0.01. Bax: Bcl‐2‐associated X protein; Bcl‐2: B‐cell leukemia‐2; PCNA: proliferating cell nuclear antigen; VEGF: vascular endothelial growth factor; MMP‐2: matrix metalloproteinases 2; MMP‐9: matrix metalloproteinases 9

Apoptosis evasion in NDEA‐induced hepatocarcinogenesis is associated with imbalance in proapoptotic and antiapoptotic proteins combined with regulation of caspases (Gupta, Bhatia, Bansal, & Koul, 2016; Subramanian & Arul, 2013). As shown in Figures 4 and 5, Diet I and Diet II significantly reduced the expression of the antiapoptotic gene Bcl‐2 at both mRNA and protein levels in rat liver tissues relative to the ConD+NDEA group (*p *<* *0.01 or *p *<* *0.05), and they increased the expression of Bax, p53, Caspase‐3, and Caspase‐8 at both the mRNA and protein levels (*p *<* *0.01 or *p *<* *0.05). These results suggest that Diet I and Diet II could induce apoptosis of NDEA‐induced HCC at the initial stages.

Apoptosis may promote cancer cell proliferation, angiogenesis, invasion, and metastasis^[41]^. As shown in Figures 4 and 5, the results of immunohistochemistry, qRT‐PCR, and Western blot showed that cell proliferation markers PCNA, angiogenic factor VEGF, and matrix metalloproteinases (MMP‐2, MMP‐9) in the liver tissue of ConD+NDEA group were all significantly higher at both the mRNA and protein levels than in the ConD group (*p *<* *0.01 or *p *<* *0.05). The expression of PCNA, VEGF, MMP‐2, and MMP‐9 in Diet I+NDEA and Diet II+NDEA groups was significantly lower than in the NDEA group (*p *<* *0.01 or *p *<* *0.05), indicating that Diet I and Diet II could inhibit cancer proliferation and affect tumor neovascularization, invasion, and metastasis.

## 4 DISCUSSION

NDEA is a potent hepatocarcinogenic nitrosamine present in a variety of foods and also a commonly used chemical carcinogen to induce hepatocellular carcinoma in animal models (Ajiboye et al., 2013). Previous studies have shown that the methods of NDEA‐induced HCC model through administration of NDEA by free drinking water, gavage, or intraperitoneal injection can simulate the real situation of HCC in human liver and represents an ideal in vivo model for evaluating prevention agents of HCC (Bishayee et al., 2011; Gupta et al., 2016). In this study, administration of the low‐dose NDEA (25 mg/kg, 2 times per week) by intraperitoneal injection for 12 weeks had succeeded in inducing liver cancer; the tumor incidence and the apparent HCC incidence were 100%, 90%, respectively, and only a low mortality rate (20%) for 20 weeks in the ConD+NDEA group.

Then we designed natural food formula rich in ACEs according to the 2007 Second Expert Report of WCRF (2007), and containing wholegrains, vegetables, fruit, and beans that fit the recommendations in the 2018 Third Expert Report (“<Summary of Third Expert Report 2018.pdf>,”), which is “similar to” the 2007 Second Expert Report (2007), but shows “a more holistic view” on the explanation of evidence on cancer prevention. Our design of food formulas is in agreement with the guidance of the new edition of WCRF by providing ACEs in the daily foods. The present investigations indicate that the Diet I and Diet II are effective chemopreventive agents against the NDEA‐induced hepatocarcinogenesis. The pathology results showed that treatment of the Diet I and Diet II had a significant effect on reducing the incidence, size, and number of hepatic nodules, and the incidence of hepatic adenoma or HCC formation in the livers of NDEA treatment rats, the low‐grade hepatic dysplasia incidence was 20% for Diet II and 40% for Diet I, the apparent HCC rates were both 0, while the apparent HCC rate of the control diet treatment rats was 90% (*p* < 0.01). The chemoprevention effects of Diet I and Diet II were superior to those of some single phytochemical or single food studied by previous studies (Bhatia, Gupta, Singh, & Koul, 2015; Gupta et al., 2016; Katayama et al., 2003). Various hepatomas exhibited high levels of AST, ALT, GGT, and AFP, as usually observed in preneoplastic and neoplastic lesions after chemical hepatocarcinogenesis (Santos, Colaco, & Oliveira, 2017). The elevated serum AST, ALT, GGT, TBIL, ALP activities and levels of AFP, TSGF, and DKK1 are indicative of poor hepatic function in the ConD+NDEA group animals compared to the ConD group animals. However, the Diet I+NDEA group and Diet II+NDEA group superior to the ConD+NDEA group showed a significant reduction in these liver function markers and tumor markers, suggesting their ability to inhibit tumor progression. Moreover, long‐term Diet I and Diet II consumption showed no toxicity in this study.

Diet plays a pivotal role in cancers prevention. Foods of plant origin are recommended, such as whole grain, vegetable, fruit, and bean, according to the WCRF (2015, 2018). Numerous epidemiological studies have shown high dietary intake of fruits and vegetables, containing phytochemicals such as resveratrol, EGCG, carotenoids, antioxidative vitamins, phenolic compounds, organosulfur compounds, flavones, isothiocyanates, indoles, and fibers could reduce cancer risk or prevent multistage carcinogenesis (Bishayee, Politis, & Darvesh, 2010; Chinni, Li, Upadhyay, Koppolu, & Sarkar, 2001; Georgia & Catherine, 2014; Gupta et al., 2016; Iriti & Varoni, 2013; Kang, Tsai, & Lee, 1999; Karen‐Ng et al., 2011; Stagos et al., 2012; Sur et al., 2016). However, there has been no report up to now on the cancer prevention effects of applying multiple food components which are derived from elite crop varieties containing high ACEs. In designing combinations, we took into account multiply plant origin foods and rich ACEs in the bred and excavated elite crop varieties. The whole grains of Job's‐tears (*Coix lacryma‐jobi* L.) variety strain 22, black rice (*Oryza sativa* L.) variety Fuzi No.2, corn, and wheat bran, etc., were used mainly for energy supply. This was complemented by the nonstarchy vegetables and fruits of various colors including *Brassica oleracea* var. *italica* FU‐1, carrot (*Daucus carota* L.) variety Y‐NS and mulberry (*Morus alba* L.) variety PR‐01, etc., and by nonsugar tea [*Camellia sinensis* (L.) O.Kuntze] variety E‐101. This is in accordance with the cancer prevention recommendation of the Third Expert Report of WCRF (2018), which emphasizes the coordination of ACEs for cancer prevention. In this study, the foods of Diet I and Diet II were produced by elite crop varieties rich in specific ACEs (Supporting Information Table S2), thus increasing concentrations of these ACEs. We observed NDEA‐induced hepatocarcinogenesis that displayed enhanced phase I enzymes and decreased phase II enzymes. This was accompanied by apoptosis evasion, enhanced cell proliferation, inflammation, invasion, and angiogenesis. Treatment of Diet I or Diet II effectively suppressed tumor incidence which was associated with modulation of liver phase I and II enzymes, accompanied by amelioration of cell proliferation, inflammation, invasion, angiogenesis, and induction of apoptosis. Thus, Diet I and Diet II appeared to affect multistage carcinogenesis related to hepatocellular carcinoma. The reasons for cancer prevention of Diet I and Diet II may include the following: (a) The food combinations containing 28 ACEs released by WCRF may exert anticancer effect at every stage of cancer processes. These 28 ACEs may also act synergistically in vivo. In particular, the foods of Diet I and Diet II were produced by elite crop varieties rich in specific ACEs (Supporting Information Table S2). (b) Daily intake of Diet I or Diet II may delay or inhibit cancer at every stage during the processes of cancer occurrence and development in a repeated fashion.

In this study, the three formulas, ConD, Diet I, and Diet II, contained the same 19 ACEs. The additional 9 unique ACEs were only included in the Diet I and Diet II. However, long‐term Diet I and Diet II consumption had no apparent HCC in Diet I+NDEA and Diet II+NDEA group, and the low‐grade hepatic dysplasia incidence was different, 20% for Diet II and 40% for Diet I, while the incidence of apparent HCC in the ConD+NDEA group was 90%. This suggests that these 9 unique ACEs are probably crucial to cancer prevention. Previous studies have also shown that the two main decomposers of glucosinolates, indoles, and isothiocyanates can inhibit the role of polyarene and nitrosamines by inhibiting cytochrome P450 isomerase (Herr & Buchler, 2010; Konsue & Ioannides, 2010; Perocco et al., 2006). EGCG, a major green tea polyphenol, has been shown to induce expression of GST, glutathione peroxidase, glutamate cysteine ligase, hemeoxygenase‐1, etc., which are involved in the elimination or inactivation of reactive oxygen species and electrophiles implicated in multistage carcinogenesis (Wang, Wang, Wan, Yang, & Zhang, 2015). Earlier reports have shown that n‐3PUFA, resveratrol, lycopene, and energy restriction may suppress phase I enzyme or enhance phase II enzyme activities to inhibit the chemical carcinogenesis (Ebert, Seidel, & Lampen, 2005; Harvie & Howell, 2016; Johnson et al., 2011; Tan et al., 2010). Numerous studies had suggested the cancer chemoprevention of organosulfur compounds may be related to its ability to inhibit phase I enzymes (such as the cytochrome P450‐dependent monooxygenases); as well as to enhance detoxification processes by inducing expression of phase II enzymes to include GST, quinone reductase, and epoxide hydrolase (Reddy, Rao, Rivenson, & Kelloff, 1993; Georgia & Catherine, 2014; Park, Kweon, & Choi, 2002; Guyonnet, Belloir, Suschetet, Siess, & Le Bon, 2001). This demonstrates that xenobiotic‐metabolizing enzymes, especially phase II enzyme are probably crucial to cancer prevention.

The metabolic activation of NDEA by cytochrome P450 enzymes produces active ethyl radical metabolites that are mainly responsible for initiation of carcinogenesis in the liver (Sadeeshkumar et al., 2017). Subsequently, reactive product of NDEA can be detoxified by phase II enzymes including GST and quinone reductase, etc. (Bishayee et al., 2011; Sindhu, Firdous, Ramnath, & Kuttan, 2013). Increased the concentrations of cytochrome P450, b5, etc. accompanied by decreased activities of GST, UGT, and EPT observed in the present study provides evidence for the initiation of carcinogenesis in ConD+NDEA group rats. Previous studies have reported that dual‐acting agents are ideal chemopreventive agents with high efficacy (De Flora et al., 2001). Our results suggest that Diet I and Diet II act as potential dual‐acting agents by suppressing phase I and enhancing phase II enzyme activity, thereby promoting detoxification and excretion. Thus, we believed that the stage “carcinogens and other environmental exposures” in Supporting Information Table S1 is the most important step responsible for these effects. During this stage, the metabolic activation of NDEA is inhibited, and its reactive products are detoxified and excreted.

In conclusion, the combinative natural food formulas Diet I and Diet II, which were produced by elite crop varieties rich in ACEs according to WCRF, both exhibited cancer preventive or delaying effects on NDEA‐induced hepatocarcinogenesis in rats, with a 0 incidence of apparent HCC (the low‐grade hepatic dysplasia incidence was 20% for Diet II and 40% for Diet I) relative to the 90% incidence of apparent HCC in the model group (*p *<* *0.01). Diet I and Diet II also can ameliorate the abnormal changes in liver function enzymes and reduce the increase in tumor markers. The results of highly significant cancer prevention or delay of Diet I and Diet II indicated excellent prospects of daily dietary for cancer prevention and the importance of the discovery and creation of ACEs‐rich crop varieties.

## CONFLICT OF INTEREST

No conflict of interest exits in the submission of this manuscript, and manuscript is approved by all authors for publication.

## ETHICAL STATEMENT

This study was approved by the Animal Ethics Committee of the Institute of Laboratory Animal Sciences at the Chinese Academy of Medical Sciences.
