# Supplementary material for: Preventive effects of combinative natural foods produced by elite crop varieties rich in anticancer effects on N‐nitrosodiethylamine‐induced hepatocellular carcinoma in rats
Source: Food Sci Nutr. 2018 Nov 29;7(1):339–55. doi: 10.1002/fsn3.896 (PMC6341211; doi:10.1002/fsn3.896)
Supplement: Supplementary file 1 [file FSN3-7-339-s001.docx]

**Preventive effects of combinative natural foods produced by elite crop varieties rich in anti-cancer effects on *N*-nitrosodiethylamine-induced hepatocellular carcinoma in rats**

**Table S1.** Anti-cancer effects on the processes underlying cell carcinogenesis and cancer development

| The carcinogenesis occurring and then progressing^A^ | | The same ACEs in ConD, Diet I and Diet II | The additional unique ACEs in the Diet I or Diet II |
| --- | --- | --- | --- |
| Normal cell | |  |  |
| Fetal exposure | |  |  |
| Body composition | |  | Energy restriction |
| Cell proliferation | | Selenium  n-3 PUFA  Flavonoids  Retinoids | Organosulfur compounds  Energy restriction  Indole-3-carbinol |
| Carcinogens,  other environmental exposures | | Zinc  Vitamin A, E, C  n-3 PUFA  Flavonoids  Selenium | Isothiocyanate  Phase II enzymes  Lycopene  Indole-3-carbinol  Organosulfur compounds  Energy restriction |
| Damaged DNA | | Folate  Coenzyme Q10  Selenium  Vitamin A |  |
| Failed apoptosis | | Genistein  Polyphenols  n-3 PUFA  Retinoids | Indole-3-carbinol |
| Differentiation | | Folate |  |
| Cell with accumulated DNA damaged and mutations Cancer potential | |  |  |
| The hallmarkers of cancer | Cell proliferation  growth signal autonomy, evasion of growth inhibitory signals, and unlimited replication | Carotenoids  Butyrate  Phytoestrogen  Daidzein  Calcium  Vitamin A (Retinoids)  n-3 PUFAs  Phenolic compounds (including genistein) | EGCG  Glucosinolate  Isothiocyanate  Energy restriction |
|  | Evasion of apoptosis | n-3 PUFAs  Genistein  Vitamin A (Retinoids)  α-tocopherol (Vitamin E)  Polyphenols | Resveratrol  Energy restriction  EGCG  Lycopene  Indole-3-carbinol  Isothiocyanate  Organosulfur compounds. |
|  | Sustained angiogenesis | Isoflavones  Quercetin  n-3 PUFAs  Flavonoids  Phytoestrogen | EGCG  Resveratrol  Energy restriction |
|  | Tissue invasion and metastasis | Quercetin  Genistein  Vitamin C, E | Resveratrol  EGCG. |
| Total no. of ACEs (without repeat) | | 19 | 9 |

A: Respectively according to the book of “Food, Nutrition, Physical Activity, and the Prevention of Cancer: a Global Perspective” in the chapter II figure 2.5 and 2.5.1—2.5.4.

ConD: control dite; ACEs: Anti-cancer effects.

**Table S2** Diet composition

| Formulas | | Flour | Fish meal | Bran | Corn | Bean cake | Dried yeast | Oleum morrhuae | Black rice | Job's-tears | Sweet potato | Broccoli | Carrot | Alfalfa | Mulberry | Reishi mushroom | Tea | Grifola frondosa | Kiwi fruit | [Pollen](javascript:;) | Trepang | Abalone | Clam | Fig | Energy restriction^a^ | No. of ACEs |
| --- | --- | --- | --- | --- | --- | --- | --- | --- | --- | --- | --- | --- | --- | --- | --- | --- | --- | --- | --- | --- | --- | --- | --- | --- | --- | --- |
| ConD | | **+** | **+** | **+** | **+** | **+** | **+** | **+** |  |  |  |  |  |  |  |  |  |  |  |  |  |  |  |  |  |  |
| Diet I | |  |  | **+** | **+** | **+** | **+** | **+** | **+** | **+** | **+** | **+** | **+** | **+** | **+** | **+** | **+** |  |  |  |  |  |  |  | **+** |  |
| Diet II | |  |  | **+** | **+** | **+** | **+** | **+** | **+** | **+** | **+** | **+** | **+** | **+** | **+** | **+** | **+** | **+** | **+** | **+** | **+** | **+** | **+** | **+** | **+** |  |
| 32 ACEs put forward  by WCRF | | | | | | | | | | | | | | | | | | | | | | | | | |  |
| 1 | Selenium | + | + | + | + | + | + |  | + | + | + | + | + | + | + | + | + | + | + | + | + | + | + | + |  | The same 19 ACEs in the ConD, Diet I, and  Diet II formulas |
| 2 | Flavonoids |  |  | + | + | + |  |  | + | + | + | + | + | + | + | + | + | + | + | + |  |  |  | + |  |  |
| 3 | Retinoids |  | + | + | + |  |  | + |  |  | + | + | + | + |  |  | + |  | + | + | + | + | + | + |  |  |
| 4 | n-3 PUFA |  | + |  |  |  |  |  |  |  |  |  |  | + | + |  |  |  |  | + | + | + | + |  |  |  |
| 5 | Zinc | + | + | + | + | + |  |  | + | + | + | + | + | + | + | + | + | + | + | + | + | + | + | + |  |  |
| 6 | Vitamin A |  | + | + | + |  |  | + |  |  | + | + | + | + |  |  | + |  | + | + | + | + | + | + |  |  |
| 7 | Vitamin C |  |  | + | + |  |  |  |  |  | + | + | + | + | + | + | + | + | + | + |  |  |  | + |  |  |
| 8 | Vitamin E |  | + | + | + | + |  |  | + | + | + | + | + | + | + | + | + | + | + | + | + | + | + | + |  |  |
| 9 | Folate |  |  | + | + | + | + |  |  |  | + | + | + | + |  |  |  | + | + |  |  |  |  | + |  |  |
| 10 | Coenzyme Q10 |  |  |  | + | + |  |  | + |  | + | + | + |  |  |  |  |  |  |  |  | + |  |  |  |  |
| 11 | Genistein |  |  |  |  | + |  |  |  |  |  |  |  | + |  |  |  |  |  |  |  |  |  |  |  |  |
| 12 | Polyphenols |  |  | + | + | + |  |  | + | + |  | + | + | + | + | + | + | + | + | + |  |  |  | + |  |  |
| 13 | Carotenoids |  |  | + | + | + |  |  |  |  | + | + | + |  |  |  | + |  |  | + |  |  |  | + |  |  |
| 14 | Butyrate | + |  | + | + |  |  |  | + | + | + |  |  |  |  |  |  |  |  |  |  |  |  |  |  |  |
| 15 | Phytoestrogen |  |  |  |  | + |  |  |  | + |  |  |  |  |  |  |  |  |  | + |  |  |  |  |  |  |
| 16 | Daidzein |  |  |  |  | + |  |  |  |  |  |  |  |  |  |  |  |  |  |  |  |  |  |  |  |  |
| 17 | Calcium | + |  | + | + | + | + |  | + | + | + | + | + | + | + | + | + | + | + | + | + | + | + | + |  |  |
| 18 | Isoflavones |  |  |  |  | + |  |  |  |  |  |  |  |  |  |  |  |  |  |  |  |  |  |  |  |  |
| 19 | Quercetin |  |  |  | + |  |  |  |  |  | + | + | + | + | + |  | + |  | + | + | + |  |  | + |  |  |
| 20 | Energy restriction |  |  |  |  |  |  |  |  |  |  |  |  |  |  |  |  |  |  |  |  |  |  |  | + | The additional unique 9 ACEs in the  Diet I or Diet II. |
| 21 | Organosulfur compounds |  |  |  |  |  |  |  |  |  |  | + |  |  |  |  |  |  |  |  |  |  |  |  |  |  |
| 22 | Indole-3- carbinol |  |  |  |  |  |  |  |  |  |  | + |  |  |  |  |  |  |  |  |  |  |  |  |  |  |
| 23 | Isothiocyanate |  |  |  |  |  |  |  |  |  |  | + |  |  |  |  |  |  |  |  |  |  |  |  |  |  |
| 24 | Phase II enzymes |  |  |  |  |  |  |  |  |  | + | + | + |  |  |  | + |  |  |  |  |  |  |  |  |  |
| 25 | Glucosinolate |  |  |  |  |  |  |  |  |  |  | + |  |  |  |  |  |  |  |  |  |  |  |  |  |  |
| 26 | EGCG |  |  |  |  |  |  |  |  |  |  |  |  |  |  |  | + |  |  |  |  |  |  |  |  |  |
| 27 | Resveratrol |  |  |  |  |  |  |  |  |  |  |  |  |  | + |  |  |  |  |  |  |  |  |  |  |  |
| 28 | Lycopene |  |  |  |  |  |  |  |  |  |  |  | + |  |  |  |  |  |  |  |  |  |  |  |  |  |
| 29 | Vanillas |  |  |  |  |  |  |  |  |  |  |  |  |  |  |  |  |  |  |  |  |  |  |  |  |  |
| 30 | Diallyl disulfide  (Garlic Extract) |  |  |  |  |  |  |  |  |  |  |  |  |  |  |  |  |  |  |  |  |  |  |  |  |  |
| 31 | Curcumin |  |  |  |  |  |  |  |  |  |  |  |  |  |  |  |  |  |  |  |  |  |  |  |  |  |
| 32 | Capsaicine |  |  |  |  |  |  |  |  |  |  |  |  |  |  |  |  |  |  |  |  |  |  |  |  |  |

Notes: “+” represents that the food contained ACEs.

^a^ The feed energies of the ConD, Diet I, and Diet II formula diets were 3.27 kcal·g^−1^, 2.63 kcal·g^−1^, and 2.60 kcal·g^−1^, respectively.

ConD: control dite; ACEs: Anti-cancer effects.

**Table S3** Primer sequences used for real-time quantitative RT-PCR and annealing temperature

| Gene symbol | Primer 1 (5′-3′) | Primer 2 (5′-3′) |
| --- | --- | --- |
| Bcl-2 | GATGACTTCTCTCGTCGCTACCGT | GGAGAAATCAAACAGAGGTCGCAT |
| Bax | CCAGGACGCATCCACCAAGAAGC | TGCCACACGGAAGAAGACCTCTCG |
| Caspase 3 | AATGAAGGGCCCTGGCACACG | GGGGAGGAGGGGTGGACTCC |
| Caspase 8 | TACTACCGAAACTTGGACC | GTGAAAGTAGGTTGTGGC |
| P53 | ACAGCAAGGATACACACAAGAAG | CCAGCACGGAGTACCAGTA |
| PCNA | CTAGCCATGGGCGTGAAC | GAATACTAGTGCTAAGGTGTCTGCAT |
| NF-κB | TGATGACATACTCCCACAAG | CAATATCCCCAGACCTAAC |
| COX-2 | ACCAGCAGTTCCAGTATCAGA | AAGTGAGCAAGTCCGTGTTC |
| TNF-α | GGAACTGGCAGAGGAGGCGC | CCCCGCCACGAGCAGGAAT |
| VEGF | ATGCGAATGTTCCTGTAAAGTGA | TAATCTGCCAAGTGGACAATCTG |
| MMP-2 | GCAACCACAACCAACTACGA | TACCAGTGTCAGTATCAGCATCA |
| MMP-9 | ATCTGTATGGTCGTGGCTCTAA | AATTGGCTTCCTCCGTGATTC |
| GAPDH | CGGAGTCAACGGATTTGGTCGTAT | AGCCTTCTCCATGGTGGTGAAGAC |

NF-κB: nuclear factor-kappa B; COX-2: cyclooxygenase-2; TNF-α: tumor necrosis factor –alpha; Bcl-2: B-cell leukemia-2; Bax: Bcl-2-associated X protein; PCNA: proliferating cell nuclear antigen; VEGF: vascular endothelial growth factor; MMP-2: matrix matalloproteinases 2; MMP- 9: matrix matalloproteinases 9.

**Table S4** Effects of Diet I and Diet II on tea/water intake, feed intake, and body weights of NDEA-induced hepatocarcinogenesis rats.

| Parameters | ConD | ConD+NDEA | Diet I+NDEA | Diet II+NDEA |
| --- | --- | --- | --- | --- |
| Body weight before (g) | 198.7±7.8 | 198.7±7.7 | 198.6±7.6 | 199.0±8.7 |
| Body weight after (g) | 587.9±55.3 | 537.9±55.9* | 482.7±39.2**^##^ | 492.2±22.9**^#^ |
| Tea/water intake (ml/100gBW/day) | 15.70± 3.04 | 11.72±4.07* | 12.26±3.39 | 13.36±2.92^#^ |
| Food intake (g/100gBW/day) | 6.46±1.64 | 5.16±1.57* | 6.15±1.34^#^ | 6.15±1.58^#^ |

ConD: normal control group; ConD+NDEA: model group, NDEA plus control diet treated group; Diet I+NDEA: NDEA plus Diet I treated group; Diet II+NDEA: NDEA plus Diet II treated group.

Values are given as mean ± SD (n = 8-10). Comparisons: compared with ConD group, *: *P* < 0.05; **: *P* < 0.01; compared with ConD+NDEA group, ^#^: *P* < 0.05; ^##^: *P* < 0.01. ConD: control dite; NDEA: *N*-nitrosodiethylamine.

**Table S5** Hematology parameters of rats fed Diet I and Diet II at weeks 20 in carcinogenicity study

| Groups | ConD | ConD+NDEA | Diet I+NDEA | Diet II+NDEA |
| --- | --- | --- | --- | --- |
| White blood count (×10^9^·L^−1^) | 4.21±1.13 | 6.25±2.67** | 4.98±1.17^#^ | 4.34±1.75^##^ |
| Red blood count (×10^12^·L^−1^) | 8.08±0.18 | 7.30±0.34** | 7.42±0.60** | 7.45±0.53** |
| Hemoglobin (g·L^−1^) | 142.0±5.0 | 130.0±9.0** | 127.0±9.0** | 138.0±8.0^#^ |
| Hematocrit (L·L^−1^) | 0.43±0.01 | 0.40±0.03** | 0.39±0.03** | 0.42±0.03 |
| Mean corpuscular volume(fL) | 53.0±1.0 | 54.0±2.0 | 53.0±1.0 | 56.0±2.0 |
| Mean corpuscular hemoglobin (pg) | 17.6±0.5 | 17.8±0.6 | 17.2±0.6 | 18.6±0.6**^#^ |
| Mean corpuscular hemoglobin  concentration (g·L^−1^) | 331.0±5.0 | 329.0±6.0 | 326.0±7.0 | 332.0±7.0 |
| Platelets (×10^9^·L^−1^) | 837.0±306.0 | 986.0±68.0 | 1012.0±108.0 | 1022.0±181.0 |
| Lymphocytes (×10^9^·L^−1^) | 2.24±0.49 | 2.73±0.67^*^ | 2.85±0.93 | 2.31±0.41^#^ |
| Lymphocytes (%) | 54.4±8.9 | 47.3±11.6 | 56.7±6.7 | 54.1±7.3 |
| Monocytes (×10^9^·L^−1^) | 0.02±0.02 | 0.04±0.04 | 0.02±0.01 | 0.02±0.01 |
| Monocytes (%) | 0.6±0.4 | 0.6±0.3 | 0.4±0.3 | 0.4±0.2 |
| Neutrophils (×10^9^·L^−1^) | 1.77±0.81 | 3.24±1.40** | 1.93±0.38^##^ | 1.87±1.00^##^ |
| Neutrophils (%) | 40.3±9.3 | 48.0±11.7 | 39.3±6.5 | 42.1±7.1 |
| Eosinophils (×10^9^·L^−1^) | 0.16±0.07 | 0.21±0.09 | 0.15±0.06 | 0.12±0.08 |
| Eosinophils (%) | 4.0±1.9 | 3.5±1.1 | 3.1±1.3 | 2.8±1.9 |
| Basophils(10^9^·L^−1^) | 0.03±0.02 | 0.04±0.02 | 0.03±0.01 | 0.03±0.02 |
| Basophils (%) | 0.7±0.5 | 0.7±0.3 | 0.6±0.2 | 0.7±0.3 |
| Reticulocyte(×10^12^·L^−1^) | 0.24±0.04 | 0.32±0.10 | 0.25±0.08 | 0.24±0.1 |
| Reticulocyte (%) | 1.5±0.2 | 2.2±0.7 | 1.7±0.6 | 1.6±0.8 |

ConD: normal control group; ConD + NDEA: model group, NDEA plus control diet treated group; Diet I+NDEA: NDEA plus Diet I treated group; Diet II+NDEA: NDEA plus Diet II treated group.

Values are given as mean ± SD (n = 8-10).Comparisons: compared with ConD group, *: *P* < 0.05; **: *P* < 0.01; compared with ConD + NDEA group, ^#^: *P* < 0.05; ^##^: *P* < 0.01.
